# Supplementary material for: Time-Dependent Effect of Eggshell Membrane on Monosodium-Iodoacetate-Induced Osteoarthritis: Early-Stage Inflammation Control and Late-Stage Cartilage Protection
Source: Nutrients. 2024 Jun 14;16(12):1885. doi: 10.3390/nu16121885 (PMC11206400; doi:10.3390/nu16121885)
Supplement: Supplementary file 1 [file nutrients-16-01885-s001.zip › nutrients-3030360-supplementary.pdf]

# Supplementary Figures

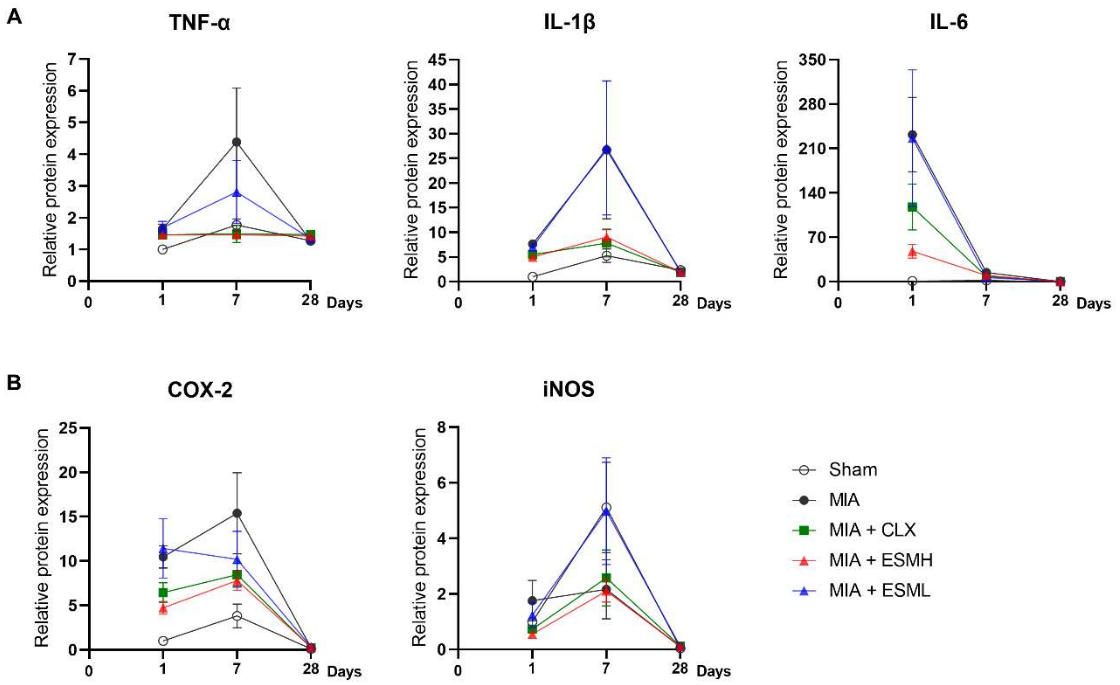


**Supplementary Figure S1.** Effect of ESM oral administration on proinflammatory cytokines and inflammatory mediators depending on the stage of disease progression in a mouse model of MIA-induced OA. Mice received daily oral administration of vehicle, CLX (positive control), ESML and ESMH, respectively. Mice cartilage tissue samples were obtained on days 1, 7, and 28 post-OA induction. The mRNA expression of pro-inflammatory cytokines including TNF-$\alpha$, IL-1β, IL-6 (**A**), and inflammatory mediators such as COX-2 and iNOS **(B**) were determined using RT-qPCR. Data were normalized to ß-actin expression levels. Data values are presented as mean ± SEM ($n$ = 6 per group).

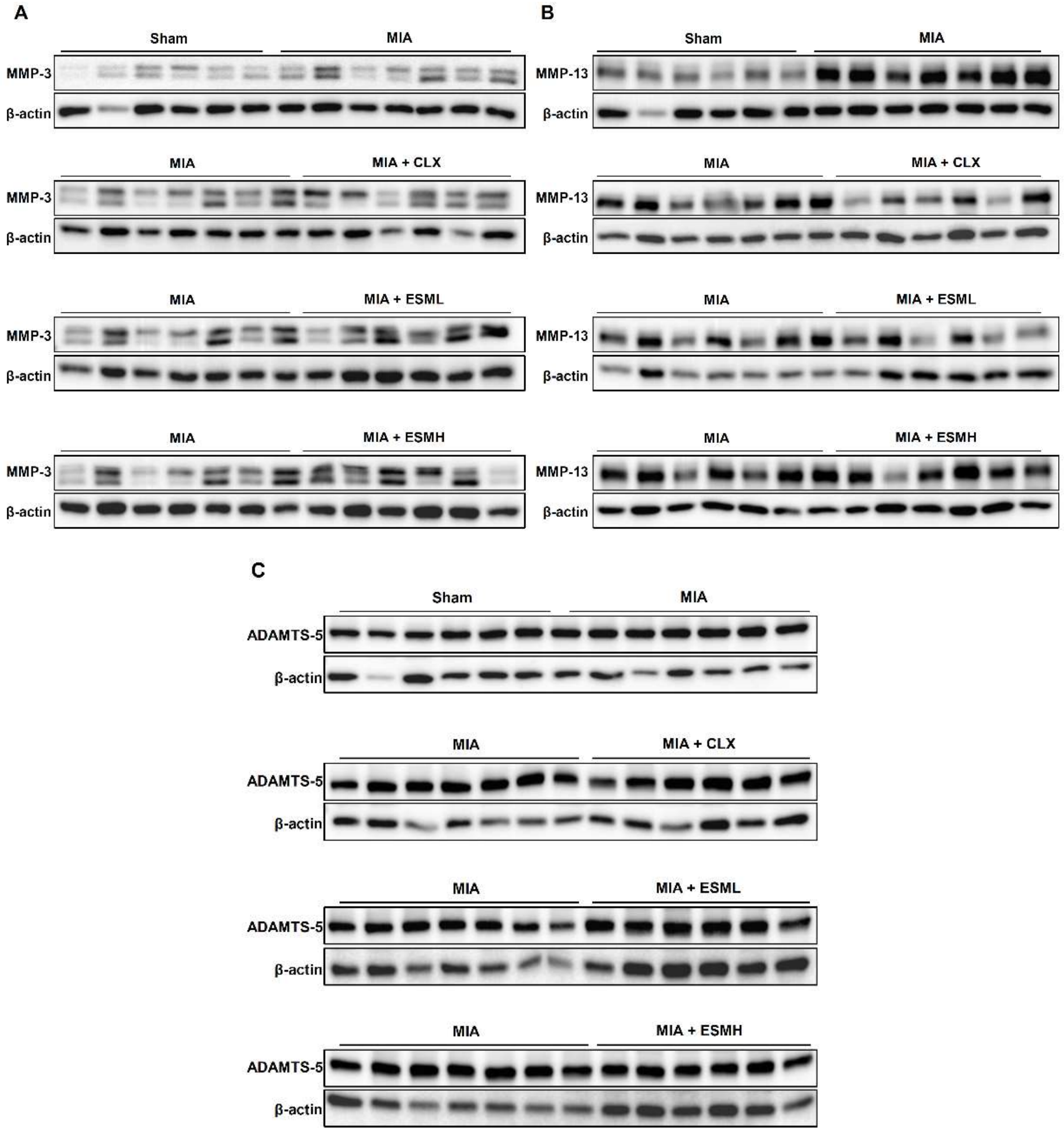


**Supplementary Figure S2.** Complete Western blots of cartilage degrading enzyme protein expression in the late stage of individual MIA-induced OA rats. Rats received daily oral administration of vehicle, CLX (positive control), ESML and ESMH, respectively. Articular cartilage tissue samples were collected on day 28 post-OA induction for protein expression analysis. Western blot analysis was performed to determined protein levels of MMP-3 (**A**), MMP-13 (**B**), and ADAMTS-5 (**C**). Protein loading was normalized to ß-actin ($n$ = 6–7 per group).

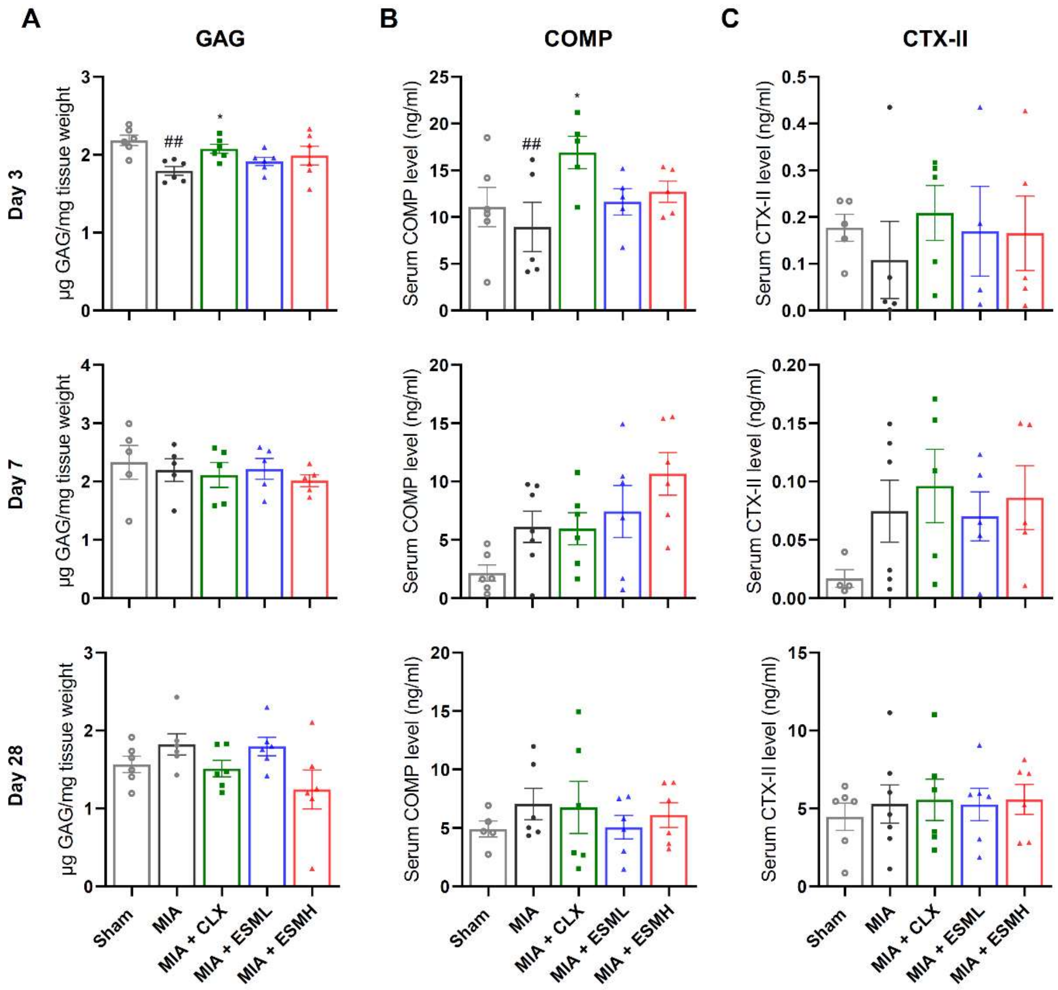


**Supplementary Figure S3.** Effect of ESM oral administration on cartilage GAG content and serum COMP, CTX-II levels in MIA-induced OA rats. Rats received daily oral administration of vehicle, CLX (positive control), ESML and ESMH, respectively. Rat cartilage tissue samples and blood were obtained on days 3, 7, and 28. (**A**) The GAG concentration was quantified using the Blyscan™ GAG assay kit (normalized to tissue weight). (**B, C**) The serum levels of COMP and CTX-II were measured using an ELISA kit. Data values are presented as mean ± SEM ($n$ = 6 per group). Statistical significance was determined using unpaired Student's $t$-test. Significant differences are indicated as follows: ## $p < 0.01$ compared to Sham; * $p < 0.05$ compared to MIA.

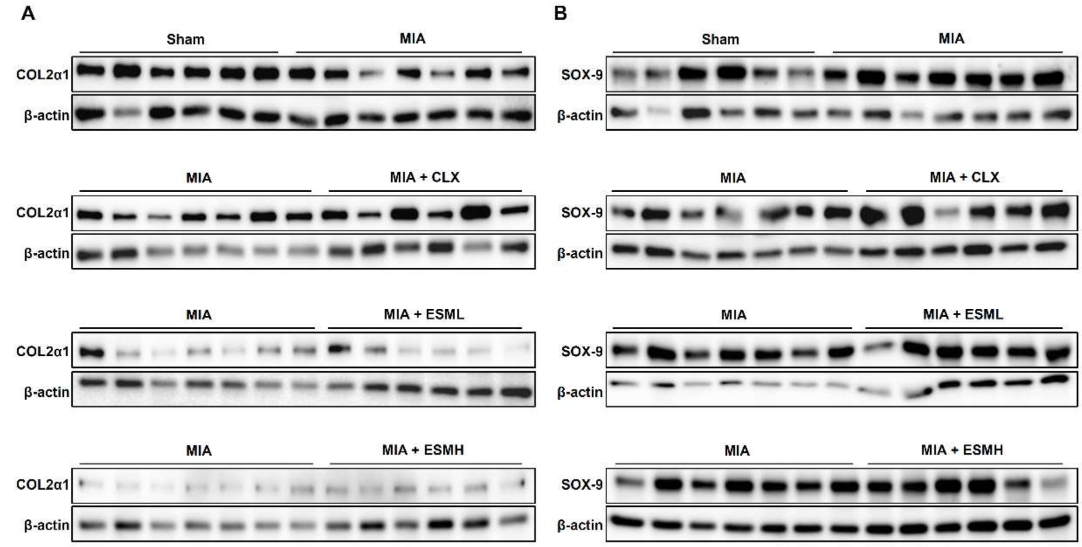


**Supplementary Figure S4.** Complete Western blots of cartilage components and synthesis transcription factor in the late stage of individual MIA-induced OA rats. Rats received daily oral administration of vehicle, CLX (positive control), ESML and ESMH, respectively. Articular cartilage tissue samples were collected on day 28 post-OA induction for protein expression analysis. Western blot analysis was performed to determined protein levels of COL2$\alpha$1 (**A**) and SOX-9 (**B**). Protein loading was normalized to ß-actin ($n$ = 6–7 per group).
